# S-Methylmethionine Attenuates Aflatoxin B1-Induced Mammary Epithelial Injury: Functional Assessment and Exploratory ATAC-Seq/RNA-Seq Analysis

**DOI:** 10.3390/toxins18080344

**Published:** 2026-08-08

**Authors:** Jin Huang, Chenxi Ling, Yuxuan Li, Yake Wang, Liyu Yang, Qiuliang Xu, Hongyu Deng, Congcong Li

**Affiliations:** 1 College of Animal Science and Technology, Henan University of Animal Husbandry and Economy, Zhengzhou 450046, China; 251001@hnuahe.edu.cn (J.H.); liyuxuan031216@163.com (Y.L.); yake2116@163.com (Y.W.); ly.yang.work@outlook.com (L.Y.); 80465@hnuahe.edu.cn (Q.X.); 2 College of Animal Sciences, Xinjiang Agricultural University, Urumqi 830052, China; c6188cc@163.com; 3 Henan Pig Bio-Breeding Research Institute, Zhengzhou 450046, China; 4 Henan Key Laboratory of Livestock and Poultry Genetic Improvement and Healthy Breeding, Zhengzhou 450046, China; 5 Henan Livestock and Poultry Genetic Resources Protection Engineering Technology Research Center, Zhengzhou 450046, China

**Keywords:** aflatoxin B1, S-methylmethionine, mammary epithelial cells, oxidative stress, ATAC-seq/RNA-seq

## Abstract

Aflatoxin 1. (AFB1) threatens animal health and dairy safety, but mammary-protective interventions remain poorly defined. We evaluated whether S-methylmethionine (SMM; commonly known as vitamin U) attenuates AFB1-associated injury using MAC-T bovine mammary epithelial cells, a mouse mammary model, biochemical assays, RT-qPCR, Western blotting, H&E staining, and exploratory ATAC-seq/RNA-seq analysis. Under 4 μg/mL (approximately 12.81 μM) AFB1 challenge, 1 mM SMM increased MAC-T cell viability by 14.1% (Tukey-adjusted *p* = 0.0387) and reduced LDH release by 38.2% (*p* = 0.000236) relative to the AFB1-only group. SMM also shifted selected redox markers toward a less injured state. Nrf2 protein abundance differed between the AF and AU groups (*p* = 0.049), although pathway activation and causal dependence were not tested. In mice, SMM co-treatment reduced serum ALT by 38.5% and mammary MDA by 56.4%, while increasing mammary T-SOD by 85.4% and GSH-Px by 100.9% relative to AF (*p* < 0.05 for the reported pairwise comparisons); inflammatory transcripts changed non-uniformly. The AU-versus-AF integration yielded four exploratory candidate genes (TNFSF10, NBEAL1, ENSBTAG00000054644, and ENSBTAG00000052086), and the selected RT-qPCR results were directionally consistent with the RNA-seq data. Thus, SMM attenuated selected AFB1-associated injury features under the tested conditions, while the underlying mechanism remains unresolved.

## 1. Introduction

Aflatoxin B1 (AFB1) is a major feed and food mycotoxin with established hepatotoxic and carcinogenic effects [1,2,3,4], as well as immunotoxic and growth-impairing effects [5,6,7] and broad disruption of cellular stress-response systems [8,9,10]. In dairy production, dietary AFB1 can be carried over into milk as aflatoxin M1 [11,12], which has been documented in bovine and ewe milk and dairy products [13,14,15,16,17]. Milk contamination is influenced by factors including feed contamination and milk yield [18] and has prompted mitigation strategies such as dietary adsorbents [19,20]. However, mammary epithelial injury and protective interventions remain less well characterized than hepatic toxicity [1,21,22,23].

Mammary epithelial integrity is important for secretory function and tissue homeostasis [24,25]. The MAC-T cell line is a well-established model of bovine mammary epithelial biology [26,27] and has been used to study toxicant-associated injury [21,22,23]. Prior AFB1 and natural-compound studies provide experimental precedent for testing protective responses in this system [23,28,29,30], but an effective SMM-based mammary intervention has not been established.

Oxidative stress is a central feature of AFB1 toxicity. AFB1 exposure can increase lipid peroxidation, impair antioxidant enzyme systems, and alter stress-responsive transcription [1,4,9,10,31]. Nrf2 and related genes such as Hmox1 and NQO1 form a biologically plausible defense axis against oxidative injury [31,32,33], but changes in these markers do not by themselves establish pathway activation or causal dependence. Inflammatory mediators including TNFα, IL1β, and IL6 may also change during injury and recovery [34,35,36], although their responses need not be uniform.

S-methylmethionine (SMM; commonly known as vitamin U) is a naturally occurring methylated sulfonium compound. Prior studies have reported cytoprotective or antioxidant-associated effects of SMM in several injury models [37,38]. These observations provide a biological rationale for testing SMM against AFB1-associated mammary epithelial injury, but they do not establish SMM-specific feed safety, physiological mammary exposure, or superiority over other interventions.

RNA-seq and ATAC-seq provide complementary views of transcription and chromatin accessibility [39,40,41,42,43]. Their integration can define broad injury-associated responses and generate candidate genes, but small or permissively filtered overlaps should be interpreted as exploratory rather than as validated regulatory mechanisms [39,40].

**Figure 1:**
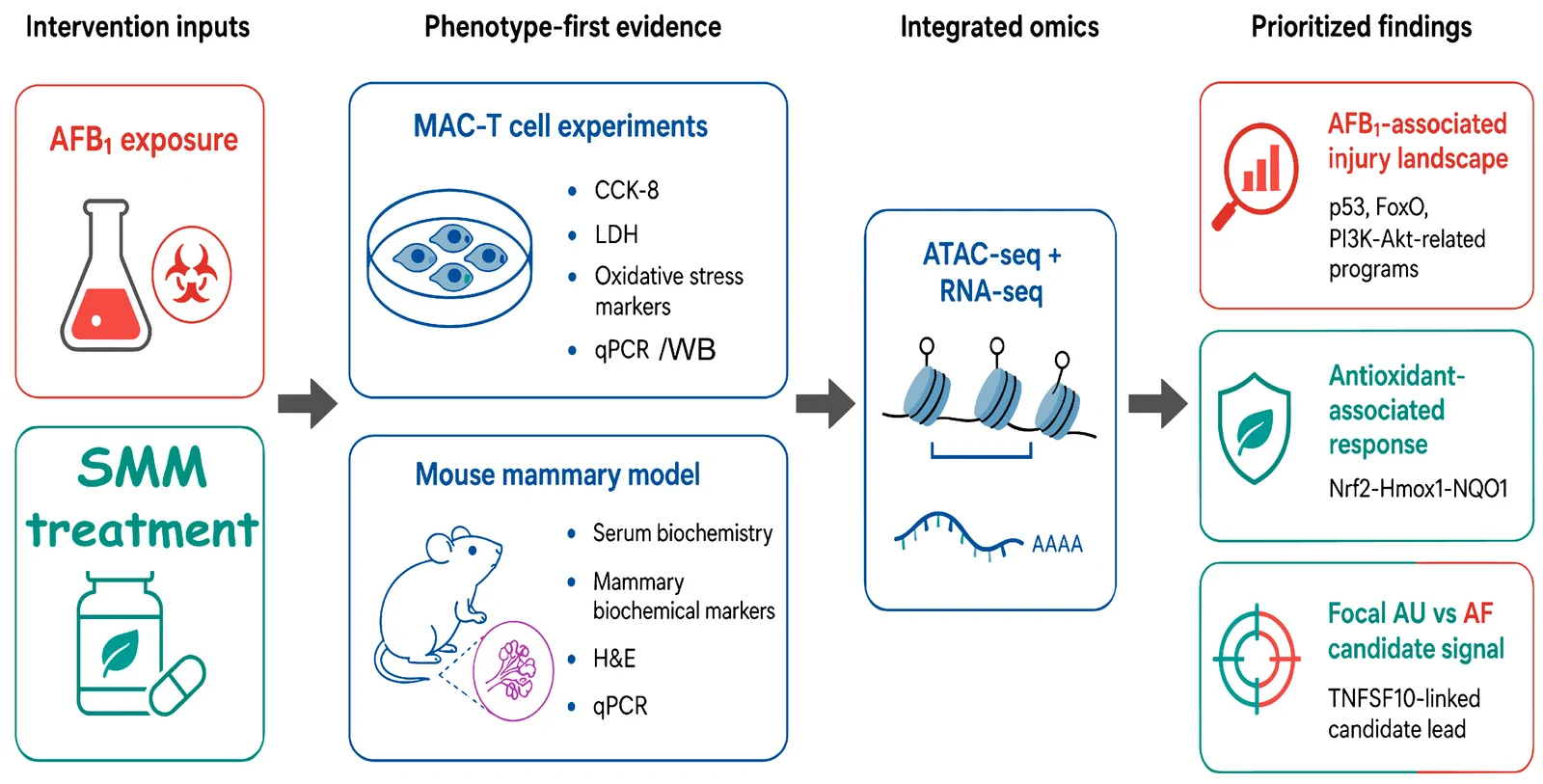
Study design and graphical summary of SMM treatment under AFB1 challenge. The workflow links MAC-T functional and biochemical assays, the mouse mammary model, and exploratory ATAC-seq/RNA-seq candidate generation. Arrows in the schematic denote the study workflow or a working hypothesis, not demonstrated molecular causality.

The objective of this study was to determine whether SMM attenuates selected AFB1-associated injury features in MAC-T cells and mouse mammary tissue and to identify molecular signals associated with that phenotype. Functional, biochemical, histological, RT-qPCR, selected protein, RNA-seq, and ATAC-seq measurements were combined for this purpose. The experimental workflow is summarized in Figure 1.

## 2. Results

### 2.1. Cell Viability and LDH Release in MAC-T Cells

**Figure 2:**
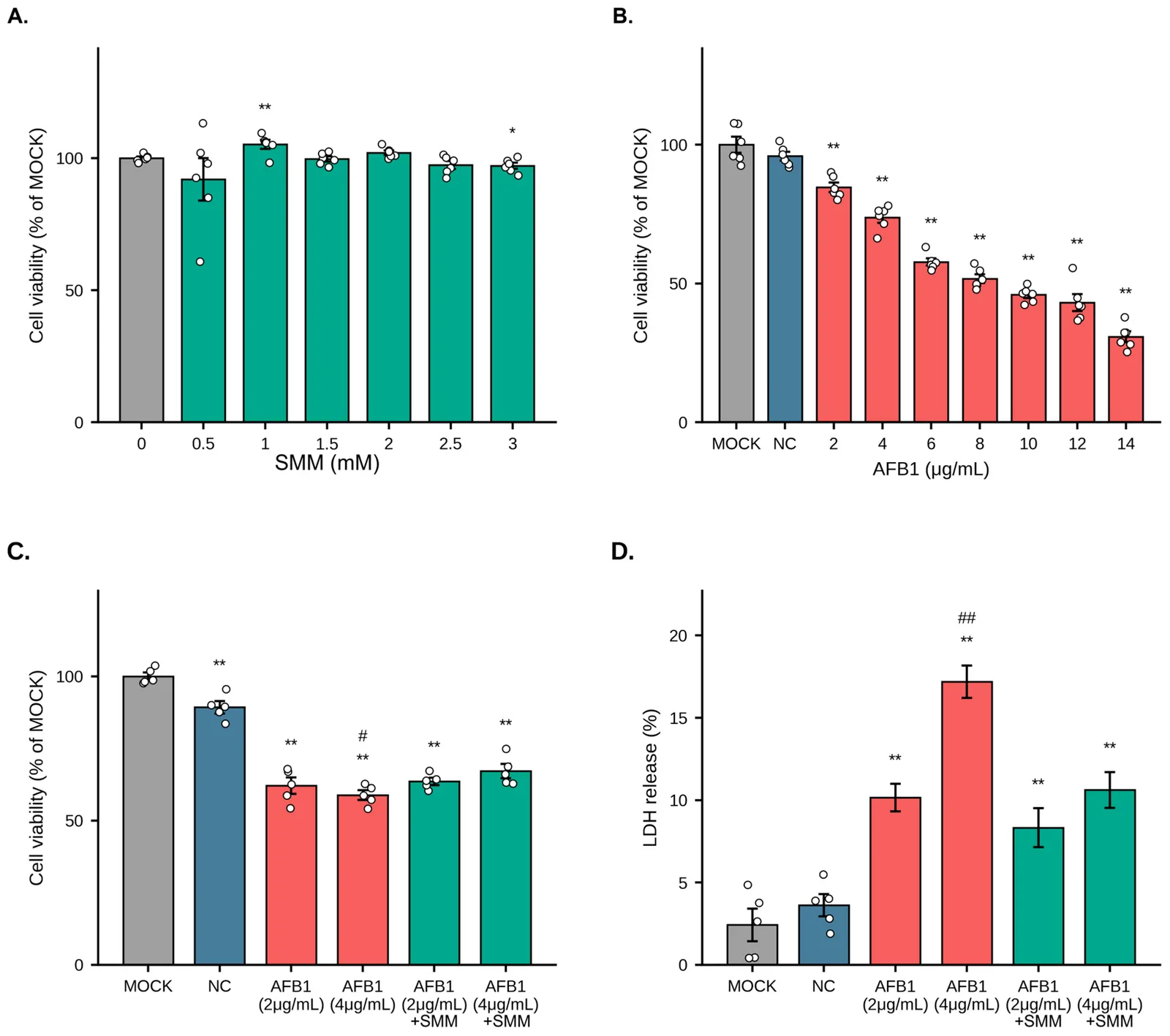
Sequential MAC-T dose screens and protection assays. (**A**) SMM cytocompatibility screen. (**B**) AFB1 dose–response. (**C**) Cell viability and (**D**) LDH release in the limited combination experiment. MOCK, untreated cells; NC, DMSO vehicle; AF, AFB1 alone; AU, AFB1 plus 1 mM SMM. The experiment was not a complete SMM-by-AFB1 factorial matrix. Panel (**A**) shows one representative experiment from three independent SMM dose-screen experiments, each of which identified 1 mM as the optimal concentration; bars show mean ± SE of six wells in the representative experiment. At 4 μg/mL (approximately 12.81 μM) AFB1, AU increased viability by 14.1% (Tukey-adjusted *p* = 0.0387) and reduced LDH release by 38.2% (Tukey-adjusted *p* = 0.000236) relative to AF. All assays were repeated in three independent biological experiments with consistent results; the plotted wells in Figure 2 represent one experiment. Open circles show the individual well-level values contributing to each mean. Significance symbols: * indicates comparison versus MOCK; # indicates the AF (4 μg/mL) versus AU (4 μg/mL + 1 mM SMM) rescue comparison. * or #, *p* < 0.05; ** or ##, *p* < 0.01.

SMM cytocompatibility and AFB1 cytotoxicity were first screened in MAC-T cells (Figure 2A,B). The SMM dose screen (0.5–3.0 mM, 24 h) was performed in three independent biological experiments. Each experiment identified 1 mM as the optimal concentration; one representative experiment is shown (1.052 ± 0.017, mean ± SE of six wells; *p* < 0.01 versus the corresponding control). Therefore, 1 mM was selected as the empirical in vitro working concentration. AFB1 was screened at 2–14 μg/mL for 24 h; the two concentrations carried forward, 2 and 4 μg/mL, correspond to approximately 6.40 and 12.81 μM, respectively, and produced sublethal reductions suitable for the protection experiment.

In the protection experiment (Figure 2C,D), 1 mM SMM was combined with 2 or 4 μg/mL AFB1 (approximately 6.40 or 12.81 μM, respectively). This was a sequential, limited combination experiment rather than a complete SMM-by-AFB1 factorial matrix; not all SMM concentrations were combined with all AFB1 concentrations. At 4 μg/mL AFB1, SMM co-treatment increased cell viability by 14.1% relative to AF (Tukey-adjusted *p* = 0.0387215) and reduced LDH release by 38.2% (Tukey-adjusted *p* = 0.000236058). The corresponding changes at 2 μg/mL AFB1 were not statistically significant. The 4 μg/mL condition was therefore used as the clearer sublethal challenge for downstream cellular assays, not as a universally optimal or physiologically representative dose.

### 2.2. Oxidative-Stress Markers, Antioxidant/Inflammatory Transcripts, and Nrf2-Axis Proteins in MAC-T Cells

**Figure 3:**
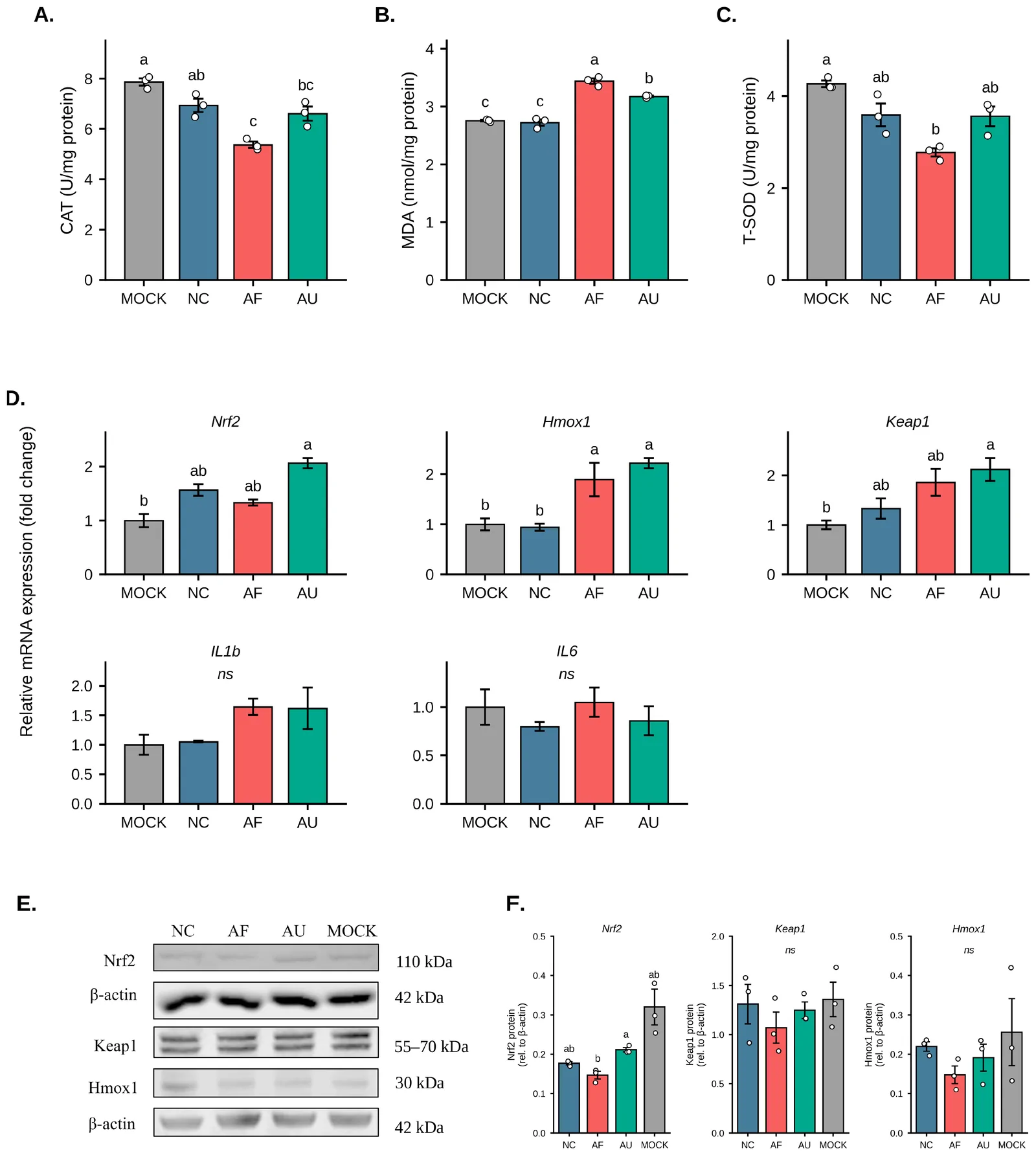
MAC-T redox, transcript, and selected protein responses. MOCK, medium replacement; NC, DMSO vehicle; AF, 4 μg/mL (approximately 12.81 μM) AFB1; AU, 4 μg/mL (approximately 12.81 μM) AFB1 plus 1 mM SMM. (**A**–**C**) CAT, MDA, and T-SOD. (**D**) Nrf2, Hmox1, Keap1, IL1β, and IL6 mRNA. (**E**) Representative immunoblots for Nrf2, Keap1, HMOX1, and β-actin. (**F**) Densitometric quantification of Nrf2, Keap1, and HMOX1 protein abundance normalized to β-actin (*n* = 3). Bars show mean ± SE, and open circles show the individual biological replicate values where displayed. Different lowercase letters above bars indicate a significant difference at *p* < 0.05; groups sharing a letter do not differ significantly; ab denotes a group that differs significantly from neither the a group nor the b group; ns indicates that no pairwise difference reached significance (*p* ≥ 0.05). The protein pattern is associative and does not demonstrate Nrf2 pathway activation.

Cellular redox status was evaluated by CAT, MDA, and T-SOD assays (Figure 3A–C). Relative to MOCK, AF showed lower CAT and T-SOD activities and higher MDA (*p* < 0.05). SMM co-treatment increased CAT and decreased MDA relative to AF (*p* < 0.05), while T-SOD shifted toward the MOCK level. Thus, the retained replicate-level evidence shows that AF, not MOCK, had the lower T-SOD activity.

RT-qPCR showed marker-specific rather than uniform responses (Figure 3D). Nrf2, Hmox1, and Keap1 were higher in AU than in MOCK (*p* < 0.05), but their AU-versus-AF differences were not significant. IL1β and IL6 did not differ significantly among the four groups; for IL1β, Welch ANOVA yielded F(3, 3.375) = 4.912 and *p* = 0.0974, and all Tamhane’s T2 comparisons were non-significant (minimum adjusted *p* = 0.2327). IL1β was numerically highest in AF. Western blotting showed an overall Nrf2 difference (Welch ANOVA *p* = 0.015), with lower Nrf2 abundance in AF than AU (Tamhane T2 *p* = 0.049). Keap1 (Kruskal–Wallis *p* = 0.557) and HMOX1 (one-way ANOVA *p* = 0.471) did not differ significantly. These findings support an Nrf2-associated observation, not demonstrated pathway activation or dependence.

### 2.3. Serum and Mammary Biochemical Markers and Mammary Transcripts in Mice

**Figure 4:**
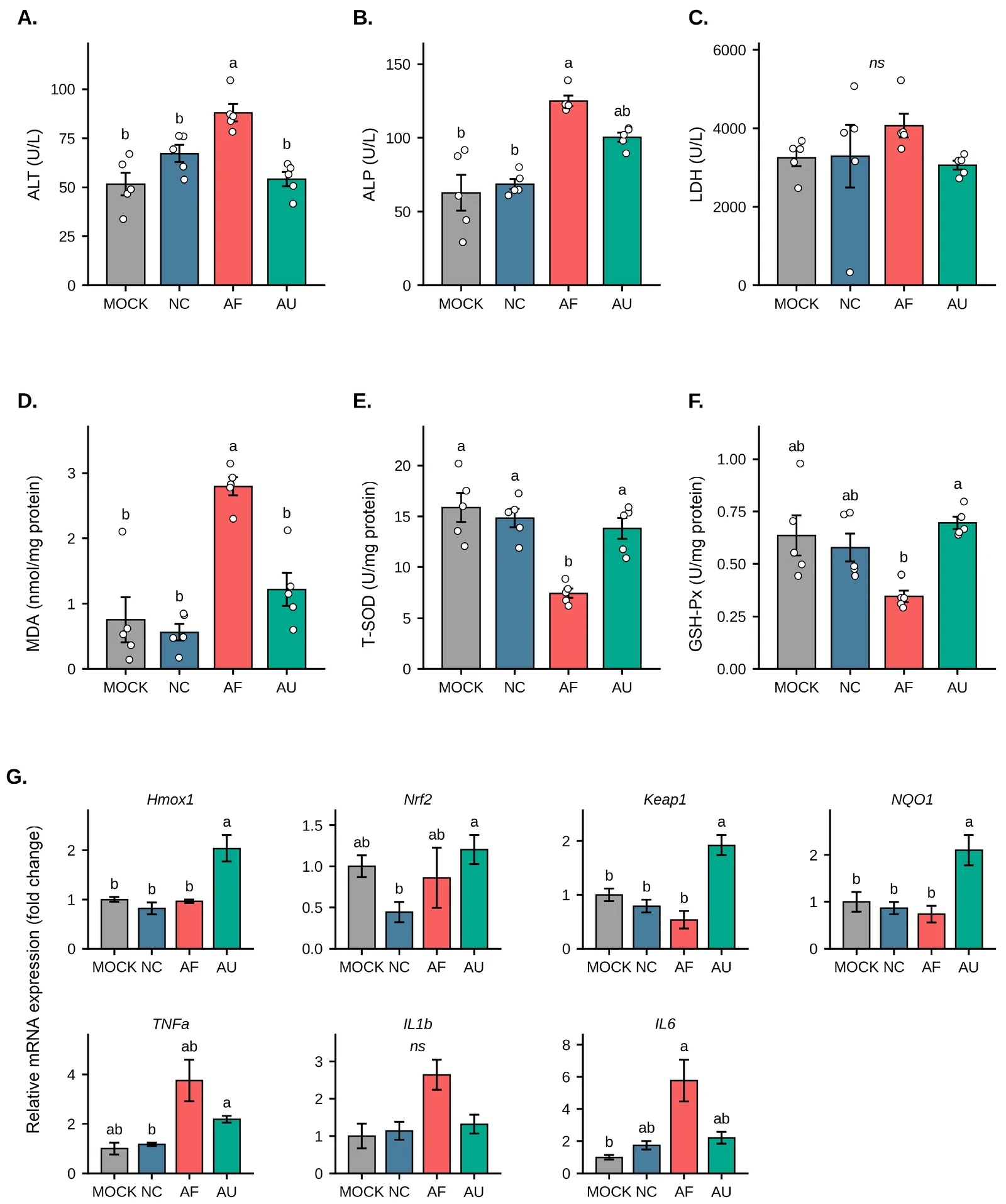
Mouse systemic, mammary biochemical, and mammary transcriptional responses. MOCK, distilled-water control; NC, DMSO vehicle; AF, 0.3 mg/kg/day AFB1; AU, 0.3 mg/kg/day AFB1 plus 50 mg/kg/day SMM. (**A**–**C**) Serum ALT, ALP, and LDH. (**D**–**F**) Mammary MDA, T-SOD, and GSH-Px (*n* = 5 dams/group); these measurements were normalized to total protein. (**G**) Mammary Hmox1, Nrf2, Keap1, NQO1, TNFα, IL1β, and IL6 mRNA (*n* = 4 dams/group). Bars show mean ± SE, and open circles show the individual animal-level values where displayed. Different lowercase letters above bars indicate a significant difference at *p* < 0.05; groups sharing a letter do not differ significantly; where shown, ns indicates that no pairwise difference reached significance (*p* ≥ 0.05). Inflammatory transcripts are interpreted marker by marker.

In mice, SMM co-treatment attenuated selected systemic biochemical changes (Figure 4A–C). Serum ALT was 38.5% lower in AU than AF (*p* < 0.05). ALP was higher in AF than MOCK and NC (*p* < 0.05), whereas AU was intermediate and did not differ significantly from AF. Serum LDH did not differ significantly among groups (Kruskal–Wallis H = 7.023, df = 3, *p* = 0.0712); all Dunn–Bonferroni pairwise comparisons were non-significant (minimum adjusted *p* = 0.0836).

Mammary redox markers shifted toward a less injured state (Figure 4D–F). Relative to AF, AU showed 56.4% lower MDA, 85.4% higher T-SOD, and 100.9% higher GSH-Px (*p* < 0.05 for the reported pairwise comparisons). These endpoint-specific results support attenuation of selected redox disturbances rather than uniform normalization of all measures.

Mammary transcript responses were non-uniform (Figure 4G). Hmox1, Nrf2, Keap1, and NQO1 were numerically higher in AU than AF, although the significant comparisons differed by marker. IL6 was higher in AF than MOCK (*p* < 0.05), IL1β did not differ significantly among groups, and TNFα was higher in AU than NC (*p* < 0.05). These data do not support a generalized anti-inflammatory conclusion.

### 2.4. Mammary Gland Index and Histopathology

**Figure 5:**
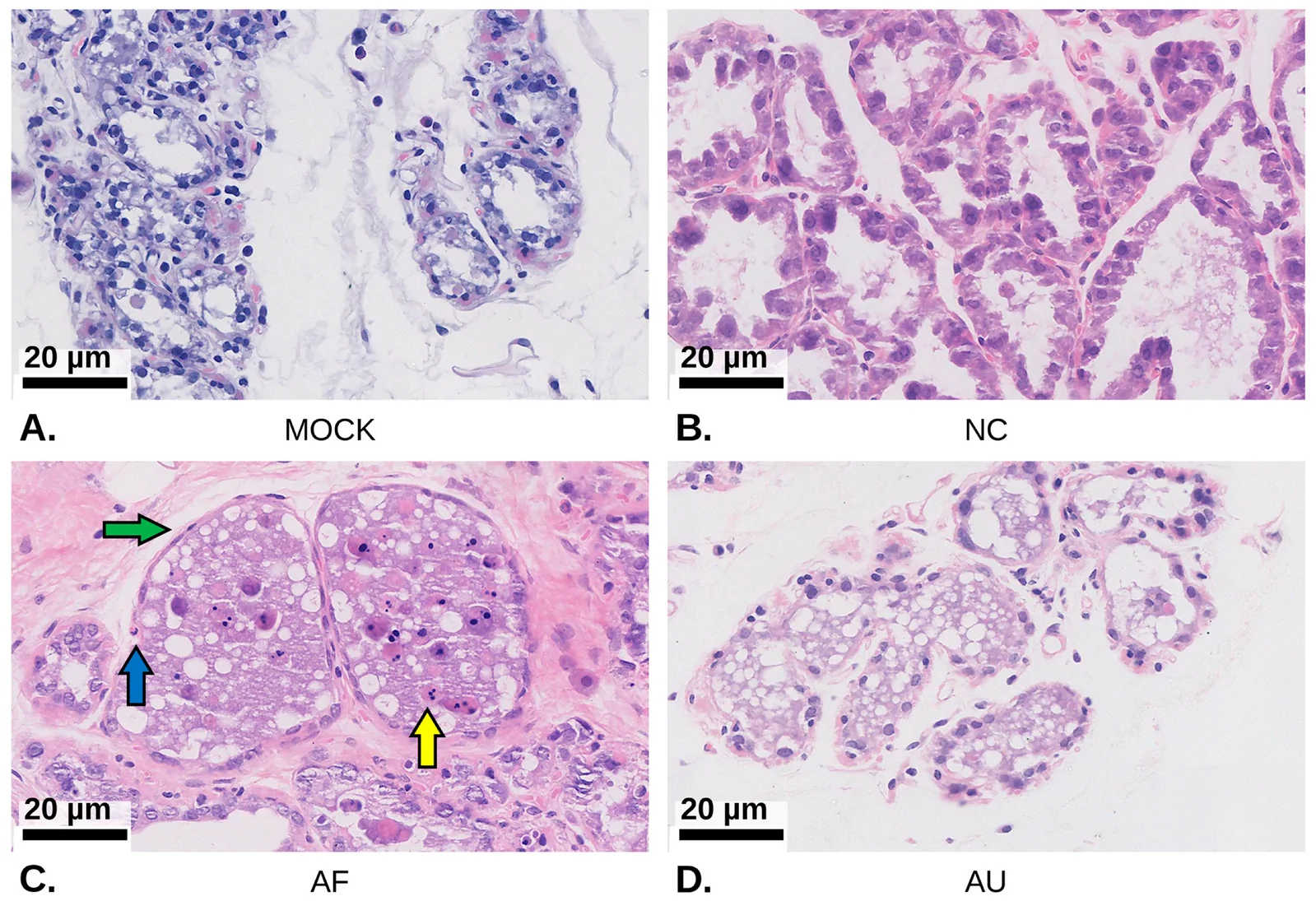
Representative mammary histology after AFB1 and SMM treatment. (**A**) MOCK, distilled-water control; (**B**) NC, DMSO vehicle; (**C**) AF, 0.3 mg/kg/day AFB1; (**D**) AU, 0.3 mg/kg/day AFB1 plus 50 mg/kg/day SMM. Scale bar: 20 μm. The green arrow indicates deformation (flattening) of mammary epithelial cells; the blue arrow indicates lymphocytes; the yellow arrow indicates cell disintegration.

The mammary gland index did not differ significantly among groups (Welch one-way ANOVA followed by Tamhane’s T2; *p* > 0.05; Supplementary Figure S4E). Representative H&E images showed relatively preserved mammary architecture in MOCK and NC, more evident epithelial deformation and reduced luminal secretion in AF, and partial morphological improvement in AU (Figure 5).

### 2.5. Integrated ATAC-Seq/RNA-Seq Defines the AFB1 Injury Landscape

**Figure 6:**
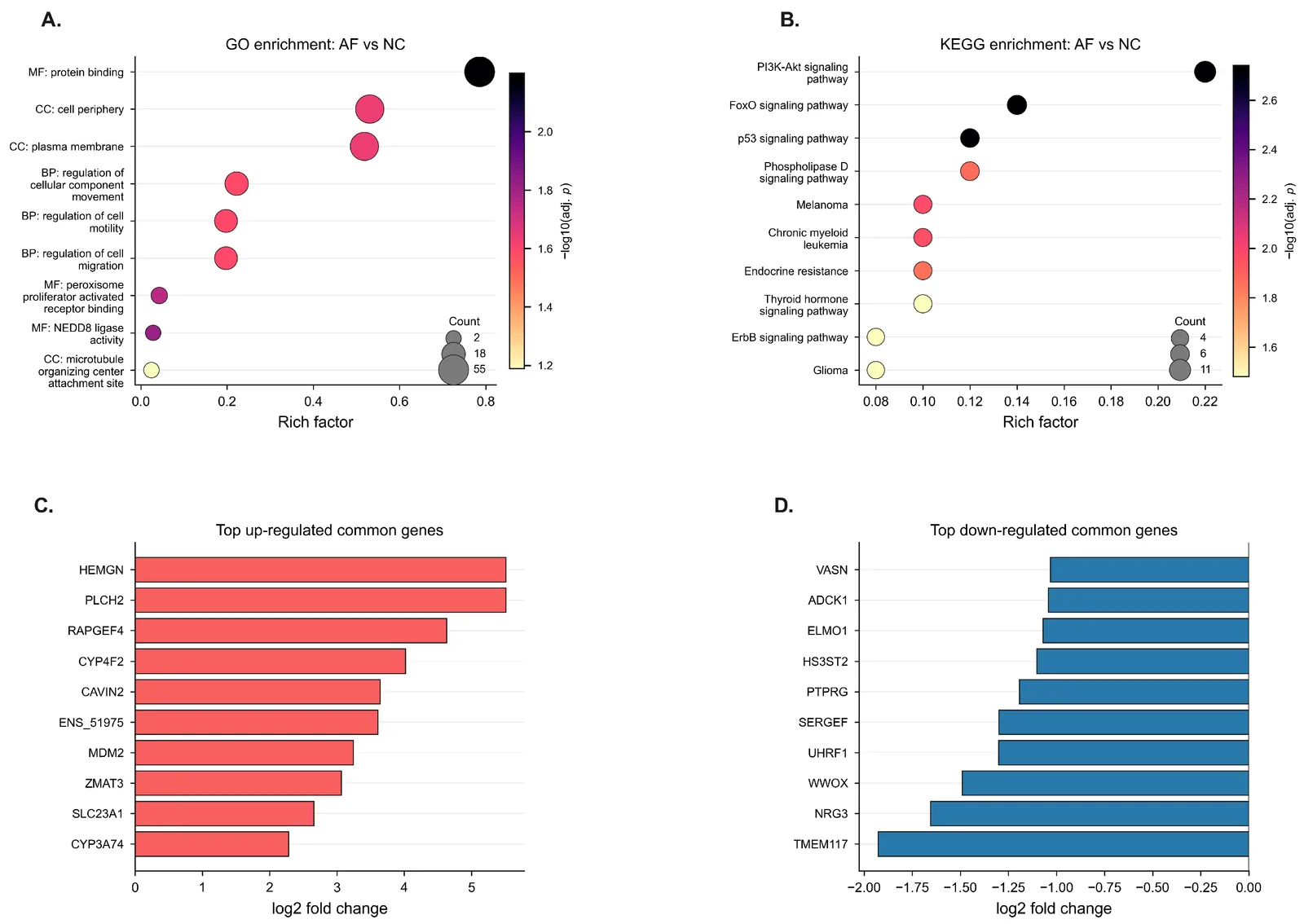
Exploratory integrated ATAC-seq/RNA-seq landscape of AFB1-associated injury. NC, DMSO vehicle; AF, 4 μg/mL (approximately 12.81 μM) AFB1. (**A**) GO enrichment for AF versus NC. (**B**) KEGG enrichment for AF versus NC. (**C**,**D**) Selected up- and down-regulated common ATAC/RNA candidate genes. Enrichment and candidate ranking are hypothesis-generating and do not establish molecular mediation.

Integrated ATAC-seq/RNA-seq analysis first characterized the broad AFB1-associated response, with AF versus NC as the primary injury comparison (Figure 6). This comparison contained 4106 differentially accessible regions (2386 increased and 1720 decreased accessibility) and 103 common ATAC/RNA genes in the retained source summary. The curated display retained 50 candidate rows. Accordingly, AF versus NC provides the main omics basis for describing an AFB1-associated injury landscape.

AF-versus-NC enrichment included membrane and regulatory GO terms and stress- and survival-related KEGG pathways, including PI3K-Akt, FoxO, and p53 signaling. Disease-labeled pathways were interpreted through shared cellular stress, proliferation, survival, or apoptosis components rather than as evidence that the corresponding diseases were modeled.

The AF-versus-NC candidate set included up-regulated genes such as HEMGN, PLCH2, RAPGEF4, CYP4F2, CAVIN2, MDM2, ZMAT3, SLC23A1, and CYP3A74 and down-regulated genes such as VASN, ADCK1, ELMO1, HS3ST2, PTPRG, SERGEF, UHRF1, WWOX, NRG3, and TMEM117. These genes are hypothesis-generating candidates within the AFB1-associated response, not validated molecular targets.

### 2.6. SMM-Associated Changes in Chromatin Accessibility and Expression

The narrower AU-versus-AF comparison was evaluated as exploratory candidate generation. Peak-to-gene assignment followed the provider’s HOMER annotation output. The 575 differentially accessible regions yielded 558 nonredundant ATAC-associated genes, which were intersected by Ensembl gene ID with 135 RNA-seq differentially expressed genes, yielding four common genes (Supplementary Figure S2). A heat map of candidate-gene expression across the twelve RNA-seq libraries, showing the row-wise Z-score of log2-transformed TPM values for each gene, is provided in Supplementary Figure S3.

The four AU-versus-AF candidates were TNFSF10, NBEAL1, ENSBTAG00000054644, and ENSBTAG00000052086. Candidate pathway annotations included cytokine–cytokine receptor interaction, apoptosis, necroptosis, FoxO signaling, and NK-cell-mediated cytotoxicity. However, their RNA-seq adjusted *p* values were approximately 0.426–0.431 under the permissive retained filter, and the apparent enrichment signal was driven by very few genes. These results therefore represent a limited exploratory signal rather than a robust SMM mechanism. Additional KEGG enrichment context for the AF-versus-MOCK, AU-versus-MOCK, AU-versus-NC, and NC-versus-MOCK comparisons is provided in Supplementary Figure S4A–D.

**Figure 7:**
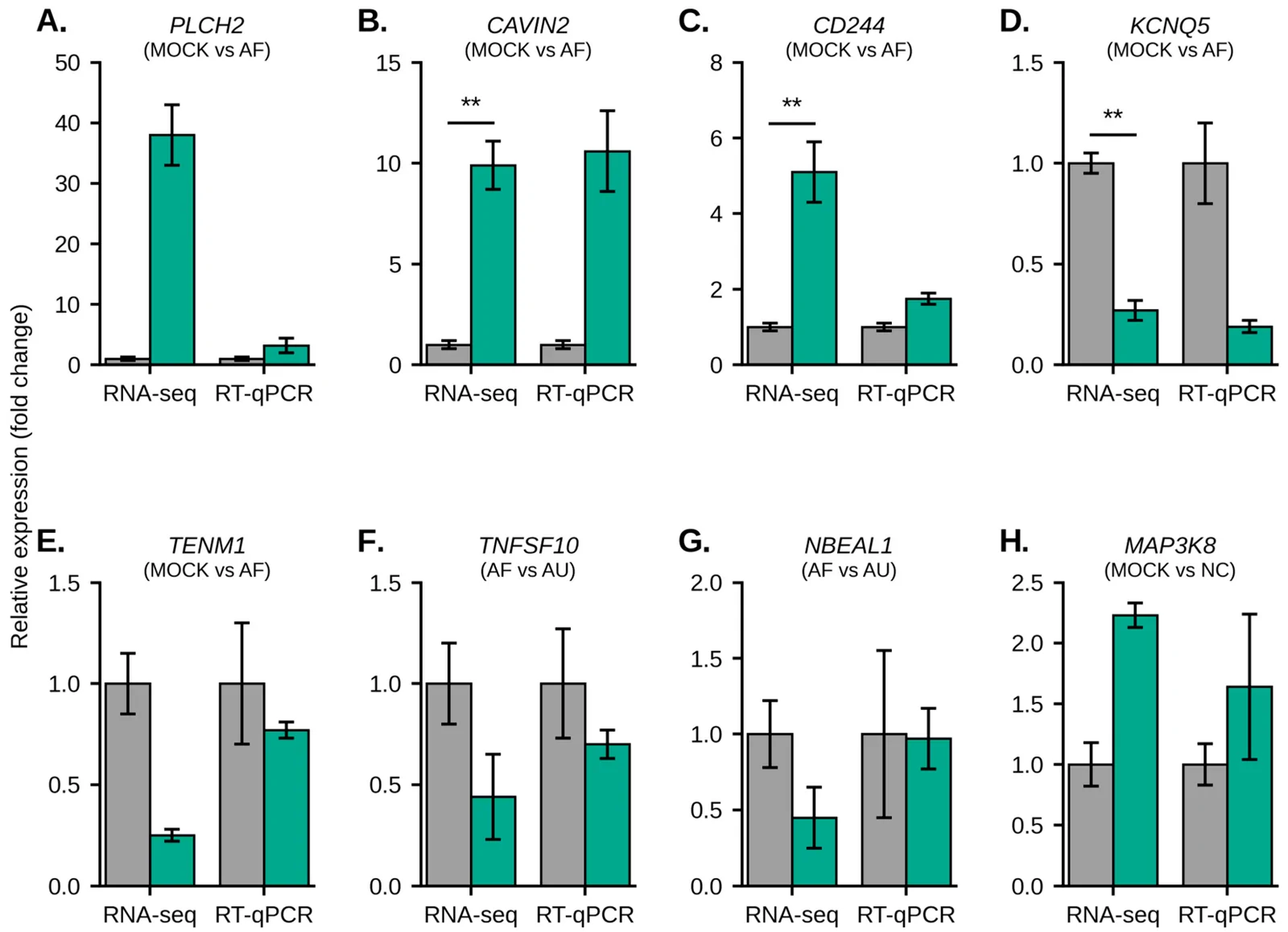
Quantitative transcript-level comparison of selected RNA-seq and RT-qPCR results. MOCK, untreated cells; NC, DMSO vehicle; AF, 4 μg/mL (approximately 12.81 μM) AFB1; AU, 4 μg/mL (approximately 12.81 μM) AFB1 plus 1 mM SMM. (**A**) *PLCH2*, (**B**) *CAVIN2*, (**C**) *CD244*, (**D**) *KCNQ5*, and (**E**) *TENM1* for the MOCK-versus-AF comparison; (**F**) *TNFSF10* and (**G**) *NBEAL1* for the AF-versus-AU comparison; and (**H**) *MAP3K8* for the MOCK-versus-NC comparison. In each panel, the left pair of bars shows the RNA-seq result and the right pair shows the RT-qPCR result. Grey bars represent the first group named in the panel heading (Group 1) and green bars represent the second group named (Group 2). Three independent biological replicates were analyzed per group, and bars show mean ± SE. Group 1 was normalized to 1.000; Group 2 therefore represents relative fold change. ** indicates *p* < 0.01 for the bracketed comparison; comparisons without an asterisk were not statistically significant. RT-qPCR directionality was consistent with RNA-seq for the eight selected genes. Exact RNA-seq raw and adjusted *p* values and RT-qPCR raw *p* values are provided in Supplementary Table S3. The panel provides transcript-level concordance and does not provide protein-level or functional validation.

**Figure 8:**
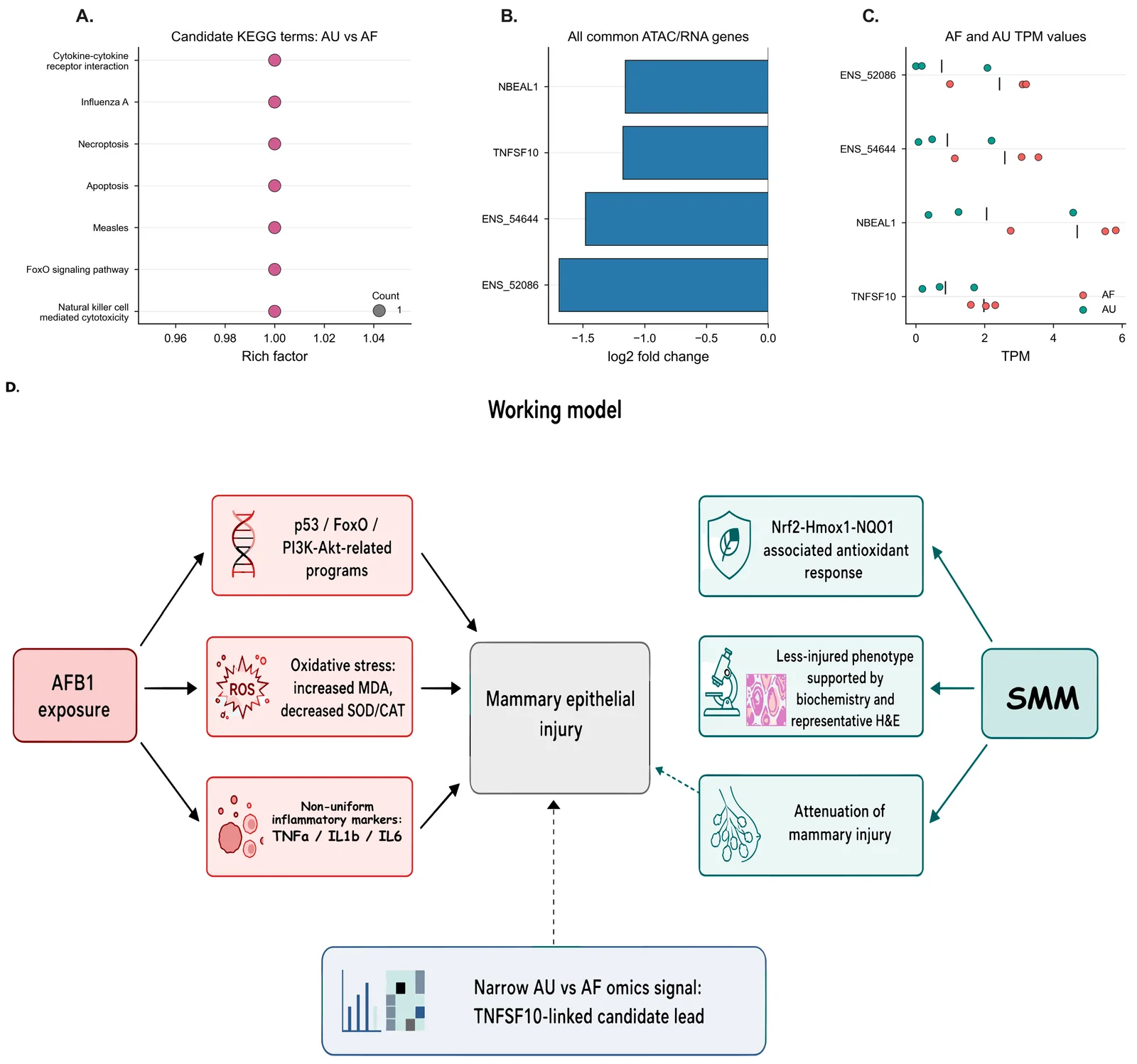
Exploratory SMM-associated candidate signals. AF, 4 μg/mL (approximately 12.81 μM) AFB1; AU, 4 μg/mL (approximately 12.81 μM) AFB1 plus 1 mM SMM. (**A**) AU-versus-AF KEGG terms under the retained permissive filter. (**B**) Four common ATAC/RNA genes. (**C**) AF and AU TPM values for the same candidates. (**D**) A working model separating the observed protective phenotype from hypothesized, functionally untested molecular links. The panel does not establish pathway activation or causal mediation. Arrows denote analytical workflow and hypothesized relationships inferred from the exploratory omics data; they do not represent experimentally demonstrated molecular causality.

RT-qPCR evaluation of eight selected genes using three independent biological replicates per group was directionally consistent with the RNA-seq data (Figure 7; Supplementary Table S3). This comparison supports transcript-level concordance and does not constitute protein-level or functional validation. The exploratory AU-versus-AF candidate signals derived from the integrated analysis are summarized in Figure 8.

## 3. Discussion

The principal finding is that SMM attenuated selected functional and biochemical features of AFB1-associated injury in MAC-T cells and mouse mammary tissue under the tested conditions. The strongest evidence is the improvement in cell viability and LDH release, selected redox measures, mouse biochemical endpoints, and representative mammary histology. Molecular measurements are interpreted as associated signals rather than proof of a causal pathway.

Redox-related changes were the most consistent biological layer. The observed CAT, T-SOD, MDA, and GSH-Px patterns are compatible with established oxidative-injury responses [44,45,46]. SMM shifted selected markers toward a less injured state. The Nrf2 protein difference between AF and AU is biologically plausible and is consistent with related bovine mammary evidence [30] and with Nrf2 as a cellular response element to mycotoxin toxicity [31] and oxidative-stress defense [45,47]; it can therefore be interpreted within the canonical Keap1-Nrf2-ARE framework [32,33] as a testable hypothesis. However, Keap1 and HMOX1 proteins did not show a coherent AF-versus-AU response, and nuclear translocation, ARE activity, and Nrf2 dependence were not tested. The Nrf2 result is therefore associative rather than causal. Inflammatory transcripts also changed non-uniformly and do not support a generalized anti-inflammatory mechanism.

The AF-versus-NC omics comparison identified a broad injury-associated accessibility and transcription landscape enriched for stress, survival, proliferation, and death-regulatory programs. These results add candidate-prioritization context to the directly measured phenotype but do not by themselves establish molecular mediation [39,40].

The AU-versus-AF result was much narrower. Only four common ATAC/RNA genes were identified under the stated exploratory thresholds, and pathway enrichment was driven by very few genes. TNFSF10 and the other candidates remain exploratory; their transcript-level directional agreement does not substitute for protein or functional validation [48,49].

Interpretation of the present findings is subject to several limitations. First, we measured Nrf2 protein abundance but did not test Nrf2 dependence, nuclear translocation, or ARE activity. The Nrf2 protein difference and selected redox changes are therefore associative and do not establish pathway activation or Nrf2-dependent protection. Second, inflammatory transcripts changed non-uniformly across markers and should be interpreted on a marker-specific basis rather than as evidence of a generalized anti-inflammatory effect. Third, the animal biochemical analyses included five dams per group, whereas mammary RT-qPCR included four dams per group. These group sizes may reduce the power to detect modest effects and increase uncertainty around the effect estimates. The animal findings warrant confirmation in larger, independent cohorts. Fourth, the AU-versus-AF integration yielded only four common ATAC-seq/RNA-seq genes. This narrow overlap, together with the absence of independent protein-level and functional validation, does not establish these candidates as mediators of SMM protection. Accordingly, these candidates remain exploratory priorities for future testing. Finally, neither MAC-T cells nor the mouse model reproduces ruminal metabolism or dairy-cow lactational physiology. The present data cannot establish ruminal stability, pharmacokinetics, mammary exposure, dairy-cow efficacy or safety, or effects on milk composition and safety. Our conclusions are therefore limited to the SMM-associated protective phenotype and redox-related changes observed under the tested cellular and mouse conditions.

Overall, the data support an SMM-associated protective phenotype under the tested cellular and mouse conditions. The measured redox-related changes and Nrf2 protein difference remain associative, inflammatory transcripts were non-uniform, and the integrated ATAC-seq/RNA-seq results nominate candidates requiring independent validation.

## 4. Conclusions

Under the tested conditions, SMM attenuated selected functional and biochemical features of AFB1-associated injury in MAC-T cells and mouse mammary tissue. The measured redox-related changes and Nrf2 protein difference did not establish pathway activation or causal dependence, inflammatory transcripts changed non-uniformly, and the integrated ATAC-seq/RNA-seq results nominated candidate signals requiring independent validation. These findings support a protective phenotype while leaving the underlying mechanism unresolved.

## 5. Materials and Methods

### 5.1. Chemicals, Reagents and Kits

AFB1 was procured from Pribolab (Qingdao, China), and SMM was purchased from Yuanye (Shanghai, China). MAC-T cells were obtained from Keycell (Wuhan, China). DMEM, fetal bovine serum, and PBS were from HyClone (Thermo Scientific, Waltham, MA, USA). CCK-8 was from GLPBIO (GK10001; Montclair, CA, USA). DMSO, trypsin-EDTA, antibiotics, Western and IP cell lysis buffer (P0013), BCA protein assay (P0010S), cellular LDH assay (C0016), MDA assay (S0131M), T-SOD assay (S0101M), CAT assay (S0051), and GSH-Px assay (S0057S) were from Beyotime Biotechnology (Shanghai, China). TRIzol (15596026CN), RevertAid First Strand cDNA Synthesis Kit (K16225), and PowerUp SYBR Green Master Mix (A25742) were from Thermo Scientific (Waltham, MA, USA). Serum ALT, ALP, and LDH reagents were from Mindray (Shenzhen Mindray Bio-Medical Electronics Co., Ltd., Shenzhen, China) and were used on the BS-240VET automated chemistry analyzer (Mindray, Shenzhen, China).

### 5.2. Cell Viability Assessment and LDH Release Evaluation in MAC-T Cells

MAC-T cells were cultured in DMEM containing 10% fetal bovine serum and the stated antibiotics at 37 °C and 5% CO_2_. All MAC-T experiments described below were independently repeated three times with consistent results. For 96-well viability and LDH assays, 1.0 × 10^4^ cells/well were used as the experiment-specific working input at the target confluence. The SMM dose screen (0.5–3.0 mM, 24 h) was performed in three independent biological experiments, each of which identified 1 mM as the optimal concentration. Figure 2A shows one representative experiment summarized from six wells per concentration. AFB1 (2–14 μg/mL) was screened separately for 24 h in six replicate wells per condition. At approximately 50–60% confluence, treatments were applied in 100 μL/well. CCK-8 reagent (10 μL/well; GK10001) was added after 24 h, incubated for 1 h, and read at 450 nm. Viability was calculated as (sample − blank)/(control − blank) × 100%.

For the protection experiment, 1 mM SMM was combined with 2 or 4 μg/mL AFB1 (approximately 6.40 or 12.81 μM, respectively). This was not a complete SMM-by-AFB1 factorial matrix; not all SMM concentrations were tested with all AFB1 concentrations. CCK-8 assays used 2% serum-containing medium, whereas LDH assays used 1% serum-containing medium. For LDH (C0016), maximum-release control reagent was added during the final hour. Supernatants were centrifuged at 400× *g* for 5 min, transferred, mixed with working reagent, incubated for 30 min in the dark, and read at 490 nm with a reference wavelength of at least 600 nm. Cytotoxicity was calculated as (treated release − spontaneous release)/(maximum release − spontaneous release) × 100%.

### 5.3. Oxidative-Stress Measurements in MAC-T Cells

For cellular redox assays, 2.5 × 10^5^ MAC-T cells/well were seeded in six-well plates as a protocol-specific working input. At 50–60% confluence, MOCK received medium replacement, NC received the DMSO vehicle, AF received 4 μg/mL (approximately 12.81 μM) AFB1, and AU received 4 μg/mL (approximately 12.81 μM) AFB1 plus 1 mM SMM. Each well received 2 mL treatment medium for 24 h.

After treatment, medium was removed and cells were washed twice with 1× PBS. For MDA and CAT, P0013 lysis buffer was added at 150 μL/well; for T-SOD, S0101M sample-preparation solution was added at 150 μL/well. Samples were kept on ice for 30 min, scraped into 1.5 mL tubes, and centrifuged at 12,000× *g* for 10 min at 4 °C. Supernatants were used for MDA (thiobarbituric-acid reaction, 532 nm), T-SOD (WST-8/xanthine-oxidase inhibition, 450 nm; 1 U was defined as 50% inhibition under the kit conditions), and CAT (residual-H_2_O_2_ colorimetry, 520 nm). BCA protein determination used a BSA standard curve at 562 nm, and enzyme activities were normalized to total protein.

### 5.4. Animal Experiment and Sample Collection

SPF-grade male and female ICR mice were acclimated for five days and mated at one male to two females per cage. A vaginal plug identified GD0.5. On GD14.5, 20 pregnant mice were randomized to MOCK, NC, AF, and AU (*n* = 5 dams/group). MOCK received 0.1 mL distilled water, NC received 0.1 mL DMSO, and AF received 0.3 mg/kg/day AFB1 by gavage based on prior mouse-exposure studies [50,51,52,53]. AU received 50 mg/kg/day SMM plus 0.3 mg/kg/day AFB1, with the SMM dose selected from prior protection studies [54,55]. Treatments were administered at 08:00 for 28 consecutive days. Natural parturition occurred at approximately GD19.5–20.5. Pups remained with their dams for normal suckling; litters were not standardized, and no early weaning, tail clipping, or ear tagging was performed. The dam was the experimental unit, and no offspring endpoint was analyzed.

Terminal collection occurred at approximately postpartum days 21–23, corresponding to late lactation near natural weaning. Dams were fasted for 12 h, weighed, and anesthetized by intraperitoneal administration of 0.3% pentobarbital sodium. Blood was collected, allowed to stand for 30 min, and centrifuged at 3000× *g* for 10 min. After cervical dislocation, mammary pairs 2–5 were collected. The left fourth gland was fixed for histology; assigned remaining tissues were snap-frozen and stored at −80 °C. Procedures were approved under HNUAHE ER 2324101.

### 5.5. Serum Biochemistry Analyses

Serum ALT, ALP, and LDH activities were measured using the corresponding Mindray reagents on a BS-240VET automated chemistry analyzer. These measurements are distinct from the Beyotime cellular LDH-release assay.

### 5.6. Mammary Gland Index and Histopathological Observation

The fourth mammary pair and fasted maternal body weight were used to calculate the mammary gland index: gland index (%) = gland weight/maternal body weight × 100%. Five dams per group were analyzed. No formal a priori sample-size equation was used.

Mammary tissues were fixed for 48 h, dehydrated through graded ethanol, cleared in xylene, paraffin-embedded, sectioned, and stained with H&E. Sections were examined at 400× magnification. The displayed images are representative observations.

### 5.7. Mammary Oxidative-Stress Assays

The third and fifth mammary pairs were used for MDA/GSH-Px and T-SOD measurements, respectively. Samples were homogenized with grinding beads at 60 Hz for 120 s twice, kept on ice for 30 min, and centrifuged at 12,000× *g* for 10 min at 4 °C. GSH-Px was measured by the DTNB/GSH-consumption method at 412 nm; MDA and T-SOD followed the principles described above. BCA total protein was used for normalization.

### 5.8. RT-qPCR

RNA was extracted using TRIzol (15596026CN), assessed using a NanoDrop spectrophotometer (Thermo Fisher Scientific, Waltham, MA, USA), and reverse transcribed with the RevertAid First Strand cDNA Synthesis Kit with DNase I (K16225, Thermo Scientific, Waltham, MA, USA). The qPCR used PowerUp SYBR Green Master Mix (A25742, Thermo Scientific, Waltham, MA, USA) on a QuantStudio 6 Pro Real-Time PCR System (Thermo Fisher Scientific, Waltham, MA, USA). Relative expression was calculated by the 2^−ΔΔCt^ method with GAPDH as the reference, following the comparative-Ct and MIQE principles [56,57]. The MAC-T RT-qPCR comparison in Figure 7 used three independent biological replicates per group, matching the three sequenced biological replicates per group; the retained two-sided unpaired equal-variance *t*-test was not changed retrospectively. Because murine mammary tissue was limited in amount and tissue from each dam was allocated to histology, oxidative-stress assays, and RT-qPCR, sufficient residual material for RT-qPCR remained for four dams per group; the remaining dam per group did not have enough residual tissue for this assay. Primer sequences are provided in Supplementary Table S1.

### 5.9. Western Blotting

Total protein was extracted from MAC-T cells using Western and IP cell lysis buffer supplemented with 1 mM PMSF (Beyotime Biotechnology). Protein concentration was determined using the BCA assay, and equal amounts of protein were separated by SDS-PAGE and transferred to membranes. Membranes were incubated with primary antibodies against Nrf2, Keap1, HMOX1, and β-actin, followed by IRDye-conjugated secondary antibodies (LI-COR Biosciences, Lincoln, NE, USA). Antibody information is provided in Supplementary Table S2. Protein bands were detected using the Odyssey DLx imaging system (LI-COR Biosciences, Lincoln, NE, USA) and quantified using ImageJ2 (version 2.14.0/1.54f; National Institutes of Health, Bethesda, MD, USA), with β-actin serving as the loading control.

### 5.10. RNA-Seq Analysis

RNA-seq included three biological samples per group and used the Bos taurus ARS-UCD1.2 genome. Library preparation, sequencing, and primary bioinformatic analysis were performed by Novogene Co., Ltd. (Beijing, China). The retained provider workflow used fastp (v0.23.2) and FastQC (v0.11.9) for quality control and HISAT2 (v2.2.1) [58] for alignment, featureCounts (Subread v2.0.1) [59] for gene-level counting, and DESeq2 (v1.36.0) [60] for differential expression. Clean-read Q30 values ranged from 96.96% to 97.35%; total mapping ranged from 97.53% to 98.35% and unique mapping from 94.74% to 95.60%. The retained provider filter was |log2 fold change| ≥ 1 with FDR < 0.05. GO and KEGG analyses used clusterProfiler (v4.4.4) [61,62,63].

### 5.11. ATAC-Seq Analysis

ATAC-seq included three biological samples per group. For each library, 5 × 10^4^ MAC-T cells were used for transposition. Following DNA purification and PCR amplification, 300–600 bp fragments were selected for sequencing.

Library preparation, sequencing, and primary bioinformatic analysis were performed by Novogene Co., Ltd. (Beijing, China). Retained provider outputs used fastp (v0.23.2)/FastQC (v0.11.9) and Bowtie2 (v2.2.4) against ARS-UCD1.2 [64], MACS2 (v2.2.7.1) peak calling [65], ChIPseeker (v1.32.1) annotation [66], HOMER (v4.11) motif and peak annotation [67], and DESeq2 (v1.36.0) differential-accessibility analysis [60]. Clean-read Q30 values ranged from 94.69% to 95.73%, total mapping from 94.33% to 98.62%, post-deduplication mapping from 66.53% to 70.85%, mitochondrial mapping from 0.21% to 0.70%, and within-group correlations from 0.88696 to 0.94165. Peaks were called at *p* < 1 × 10^−5^; differential regions were screened using |log2 fold change| > 0.585 with unadjusted *p* < 0.05. The assay followed the established ATAC-seq framework [41], and enrichment analyses used clusterProfiler (v4.4.4) [61,62,63]. Fragment-size periodicity, TSS-enrichment, PCA, and IDR profiles were reviewed in the provider quality-control report (Supplementary Figure S1).

### 5.12. ATAC-Seq/RNA-Seq Integration and Enrichment Analysis

Peak-to-gene assignment followed the provider’s HOMER annotation output. The resulting ATAC peak-associated gene set was deduplicated and intersected with RNA-seq differentially expressed genes by Ensembl gene ID. For AU versus AF, 575 differentially accessible regions yielded 558 nonredundant ATAC-associated genes; intersection with 135 RNA-seq genes yielded four common genes. This integrated analysis was used for exploratory candidate generation.

### 5.13. Statistical Analysis

Data are presented as mean ± SE. Shapiro–Wilk and Levene tests guided endpoint-specific analysis. Data meeting normality and homoscedasticity were analyzed by one-way ANOVA with Tukey tests; non-normal endpoints used Kruskal–Wallis with Bonferroni-adjusted pairwise comparisons; and normal but heteroscedastic endpoints used Welch ANOVA with Tamhane T2. MAC-T T-SOD and mouse ALP, IL6, and IL1β used the Kruskal–Wallis route. MAC-T Nrf2 and mouse mammary gland index, GSH-Px, and TNFα used Welch/Tamhane T2. MAC-T Keap1 and mouse Nrf2 used ANOVA/Tukey. Figure 7 used the retained two-sided unpaired equal-variance *t*-test. For current MAC-T IL1β, Welch ANOVA calculated from the retained group means, SE values, and *n* = 3 per group yielded F(3, 3.375) = 4.912 and *p* = 0.0974; all Tamhane’s T2 comparisons were non-significant (minimum adjusted *p* = 0.2327). Mouse serum LDH was analyzed using the retained dam-level values by Kruskal–Wallis testing (H = 7.023, df = 3, *p* = 0.0712), followed by Dunn–Bonferroni comparisons, all of which were non-significant (minimum adjusted *p* = 0.0836). Exact *p* values are reported where linked to retained endpoint-level data or reproducible summary-statistic calculations. No formal a priori sample-size calculation was performed; retained biochemical endpoints used *n* = 5 dams/group, mammary qPCR used *n* = 4/group, and Figure 7 used *n* = 3 independent biological replicates/group. All statistical analyses were performed using IBM SPSS Statistics (version 26.0; IBM Corp., Armonk, NY, USA). Sequencing analyses used the stated provider thresholds and false-discovery-rate principles [68].

## Data Availability

The RNA-seq and ATAC-seq datasets were generated in the present study; library preparation, sequencing, and primary bioinformatic analysis were performed by Novogene Co., Ltd. (Beijing, China). The datasets are publicly available in the NCBI BioProject database under accession numbers PRJNA1455733 and PRJNA1457560, respectively. Processed figure-input tables, Western blot quantification data, figure configuration files, source-data tables, and candidate-gene tables are provided as Supplementary Materials or are available from the corresponding authors upon reasonable request.

## Supplementary Materials

The following supporting information can be downloaded at: https://www.mdpi.com/article/10.3390/toxins18080344/s1, Supplementary Figure S1. ATAC-seq sequencing and library-quality overview; Supplementary Figure S2. Cross-comparison summary of the ATAC-seq and RNA-seq results; Supplementary Figure S3. Heat map of candidate-gene expression across the twelve RNA-seq libraries; Supplementary Figure S4. Exploratory KEGG enrichment context and mammary gland index; Supplementary Table S1. Primer information for qPCR; Supplementary Table S2. List of antibodies used for Western blot analysis; Supplementary Table S3. Quantitative comparison of RNA-seq and RT-qPCR results for genes shown in Figure 7; Figure 7 Ct-level source data; and Western blot quantification source data.
