# Supplementary material for: S-Methylmethionine Attenuates Aflatoxin B1-Induced Mammary Epithelial Injury: Functional Assessment and Exploratory ATAC-Seq/RNA-Seq Analysis
Source: Toxins (Basel). 2026 Aug 8;18(8):344. doi: 10.3390/toxins18080344 (PMC13517378; doi:10.3390/toxins18080344)
Supplement: Supplementary file 1 [file toxins-18-00344-s001.zip › toxins-4440111-supplementary-proof author-DONE.pdf]

Supplementary Materials: S-Methylmethionine Attenuates Aflatoxin B1-Induced Mammary Epithelial Injury: Functional Assessment and Exploratory ATAC-seq/RNA-seq Analysis

Jin Huang, Chenxi Ling, Yuxuan Li, Yake Wang, Liyu Yang, Qiuliang Xu, Hongyu Deng and Congcong Li

Supplementary Table S1. Primer information for qPCR.

**Gene**	**Primer sequence (5′–3′)**
bos-Nrf2-F	CCCAGTCCAACCTTTGTCGT
bos-Nrf2-R	TGGAGAGCTTTTGCCCGTAG
bos-Keap1-F	CTGTCCTCAACCGTCTGCTC
bos-Keap1-R	ATCCGCCACTCGTTTCTCTC
bos-Hmox1-F	GCATGCCCCAGGATTTGTCA
bos-Hmox1-R	GACGCCATCACCAGCTTAAAAC
bos-IL1β-F	AACGTCCTCCGACGAGTTTC
bos-IL1β-R	TGCAGAACACCACTTCTCGG
bos-IL6-F	GGGTTCAATCAGGCGATTTG
bos-IL6-R	CCCACTCGTTTGAAGACTGC
bos-PLCH2-F	CTATCGGCACGTGTACCTGG
bos-PLCH2-R	GAGCCTGCTTGACCTTACCA
bos-CAVIN2-F	GAGAAGTCGCGCAAGGTTAG
bos-CAVIN2-R	AGGGATCTCATTTTCTTCCTGG
bos-CD244-F	TGAGCATCGTGATTCTCCTCG
bos-CD244-R	ACTTTTGGGCTTGTCCGTGA
bos-KCNQ5-F	CCTCATTCAGTGCGTTTGGC
bos-KCNQ5-R	AGCTGAGCTTCTGGTTGGTG
bos-TENM1-F	CTTAGATGCTGTCCGAGATGGC
bos-TENM1-R	ACTCCAACCCACCTGACACA
bos-TNFSF10-F	GGGCAGCTGGAAGAAATTTATCTG
bos-TNFSF10-R	GCAGCATCACTTGGATGGTT
bos-NBEAL1-F	GTTCCCATGGGCCTAGCATC
bos-NBEAL1-R	AGCCAAATTGGGAATGGTATGT
bos-MAP3K8-F	AGACGCCGACCTCAAGAATC
bos-MAP3K8-R	ATCGGAGCCAATGTTCCTGT
bos-GAPDH-F	AGCCGTAACTTCTGTGCTGT
bos-GAPDH-R	ATGGCGACGATGTCCACTTT
mmu-Nrf2-F	ACTACAGTCCCAGCAGAGTGAT
mmu-Nrf2-R	TCACACATTTCTGCGTGCT
mmu-Keap1-F	GATGGGCAGGACCAGTTGAA
mmu-Keap1-R	CCGGGTCATAGCATTCCACA
mmu-Hmox1-F	CAGAAGAGGCTAAGACCGCC
mmu-Hmox1-R	CTCTGACGAAGTGACGCCAT
mmu-NQO1-F	GGTAGCGGCTCCATGTACTC
mmu-NQO1-R	CCAGACGGTTTCCAGACGTT
mmu-TNFα-F	CCCTCACACTCAGATCATCTTCT
mmu-TNFα-R	GCTACGACGTGGGCTACAG
mmu-IL6-F	TAGTCCTTCCTACCCCAATTTCC
mmu-IL6-R	TTGGTCCTTAGCCACTCCTTC
mmu-IL1β-F	TGCCACCTTTTGACAGTGATG
mmu-IL1β-R	ATGTGCTGCTGCGAGATTTG

mmu-GAPDH-F	ATGACTCCACTCACGGCAAA
mmu-GAPDH-R	CGGCCTCACCCCATTTGATG

Supplementary Table S2. List of antibodies used for Western blot analysis.

Antibody	Isotype	Dilution	Source	Cat. No.	Molecular weight	RRID	Class
Primary antibody							
Nrf2	Rabbit	1:500	Proteintech (Wuhan, China)	16396-1-AP	110 kDa	AB_2782956	polyclonal
Keap1	Rabbit	1:1000	Proteintech (Wuhan, China)	10503-2-AP	70 kDa	AB_2132625	polyclonal
Hmox1	Rabbit	1:1000	Proteintech (Wuhan, China)	10701-1-AP	28–33 kDa	AB_2118685	polyclonal
β-actin	Mouse	1:1000	Proteintech (Wuhan, China)	66009-1-Ig	42 kDa	AB_2687938	monoclonal
Secondary antibody							
IRDye 800CW	Goat anti-rabbit	1:10,000	LI-COR Biosciences (Lincoln, NE, USA)	—	—	—	—
IRDye 680RD	Goat anti-mouse	1:10,000	LI-COR Biosciences (Lincoln, NE, USA)	—	—	—	—

Supplementary Table S3. Quantitative comparison of RNA-seq and RT-qPCR results for genes shown in Figure 7.

Gene	Comparison (Group 2 / Group 1)	RNA-seq			RT-qPCR		Concordance assessment
		Relative expression (Group 1 = 1.000; mean ± SE)	Raw p	Adjusted p	Relative expression (Group 1 = 1.000; mean ± SE)	Raw p	
PLCH2	AF/MOCK	MOCK: 1.000 ± 0.265 AF: 38.277 ± 5.102	7.22×10^{-56}	1.37×10^{-53}	MOCK: 1.000 ± 0.350 AF: 3.566 ± 1.110	0.0921	Directional concordance; RT-qPCR NS
CAVIN2	AF/MOCK	MOCK: 1.000 ± 0.206 AF: 9.899 ± 1.293	6.07×10^{-69}	1.82×10^{-66}	MOCK: 1.000 ± 0.092 AF: 10.615 ± 1.972	0.00821	Significant concordance
CD244	AF/MOCK	MOCK: 1.000 ± 0.035 AF: 5.183 ± 0.783	5.32×10^{-36}	4.81×10^{-34}	MOCK: 1.000 ± 0.056 AF: 1.755 ± 0.127	0.0056	Significant concordance
KCNQ5	AF/MOCK	MOCK: 1.000 ± 0.055 AF: 0.270 ± 0.045	8.27×10^{-7}	6.47×10^{-6}	MOCK: 1.000 ± 0.147 AF: 0.200 ± 0.025	0.00584	Significant concordance
TENM1	AF/MOCK	MOCK: 1.000 ± 0.140	1.50×10^{-12}	2.67×10^{-11}	MOCK: 1.000 ± 0.313	0.52	Directional concordance;

		AF: 0.245 ± 0.026			AF: 0.778 ± 0.033		RT-qPCR NS
TNFSF10	AU/AF	AF: 1.000 ± 0.191<br>AU: 0.443 ± 0.215	0.1	0.426	AF: 1.000 ± 0.260<br>AU: 0.704 ± 0.058	0.329	Directional concordance; both NS
NBEAL1	AU/AF	AF: 1.000 ± 0.222<br>AU: 0.448 ± 0.266	0.112	0.426	AF: 1.000 ± 0.532<br>AU: 0.968 ± 0.208	0.958	Directional concordance; both NS
MAP3K8	NC/MOCK	MOCK: 1.000 ± 0.189<br>NC: 2.230 ± 0.106	4.31×10^{-6}	1.74×10^{-4}	MOCK: 1.000 ± 0.161<br>NC: 1.646 ± 0.575	0.34	Directional concordance; RT-qPCR NS

Notes:

1. Values are presented as mean ± SE; $n = 3$ independent biological replicates per group.

2. Relative expression was normalized to Group 1 = 1.000. Therefore, the Group 2 mean directly represents the fold change relative to Group 1; separate fold-change columns were omitted to avoid redundancy.

3. RNA-seq raw *p* value is the nominal DESeq2 *p* value; the adjusted *p* value is the multiple-testing-corrected *p* value.

4. RT-qPCR expression was calculated using the $2^{-\Delta\Delta Ct}$ method with *GAPDH* as the reference gene. Raw *p* values were obtained using a two-sided unpaired Student's *t*-test assuming equal variances.

5. NS, not significant ($p \geq 0.05$). "Significant concordance" indicates directional agreement with $p < 0.05$ by RT-qPCR; directional concordance alone does not imply protein-level or functional validation.

Supplementary Figures

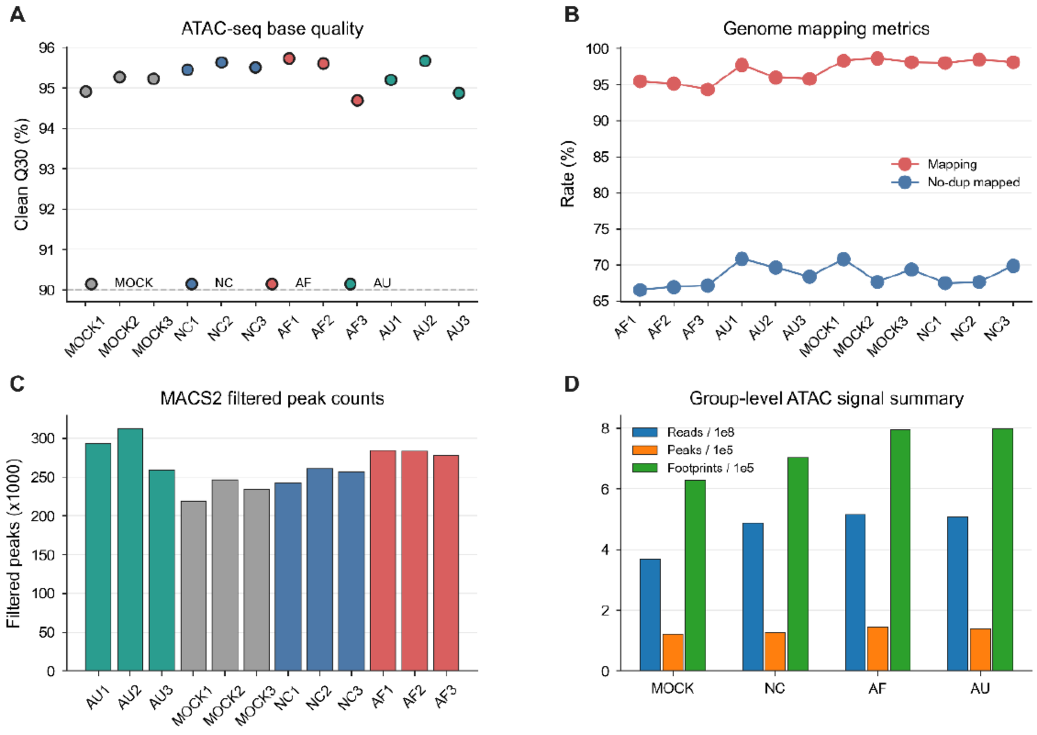

Supplementary Figure S1. ATAC-seq sequencing and library-quality overview. (A) Clean Q30 base-quality percentage of each ATAC-seq library; colours denote the four groups. (B) Genome mapping rate and non-duplicate mapped rate of each library. (C) Number of MACS2 filtered peaks per library. (D) Group-level summary of sequencing reads, filtered peaks and footprints, scaled as indicated in the legend. MOCK, untreated control; NC, solvent control; AF, AFB1 alone; AU, AFB1 combined with SMM; *n* = 3 libraries per group.

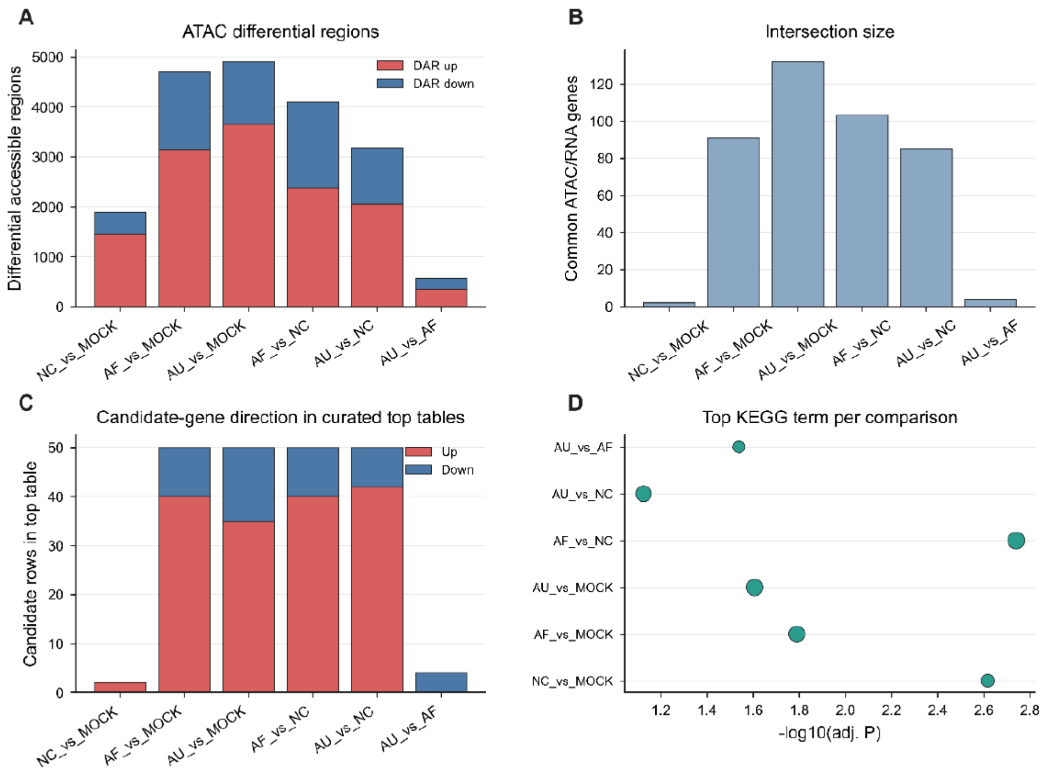

Supplementary Figure S2. Cross-comparison summary of the ATAC-seq and RNA-seq results. (A) Number of differentially accessible regions per comparison, separated into increased (DAR up) and decreased (DAR down) accessibility. (B) Number of genes shared between the ATAC-seq and RNA-seq results for each comparison. (C) Direction of change of the candidate genes listed in the curated top tables. (D) Most strongly enriched KEGG term per comparison, plotted as $-\log_{10}$(adjusted p). Comparisons are labelled as group pairs, and all results are exploratory and were not used to establish causality.

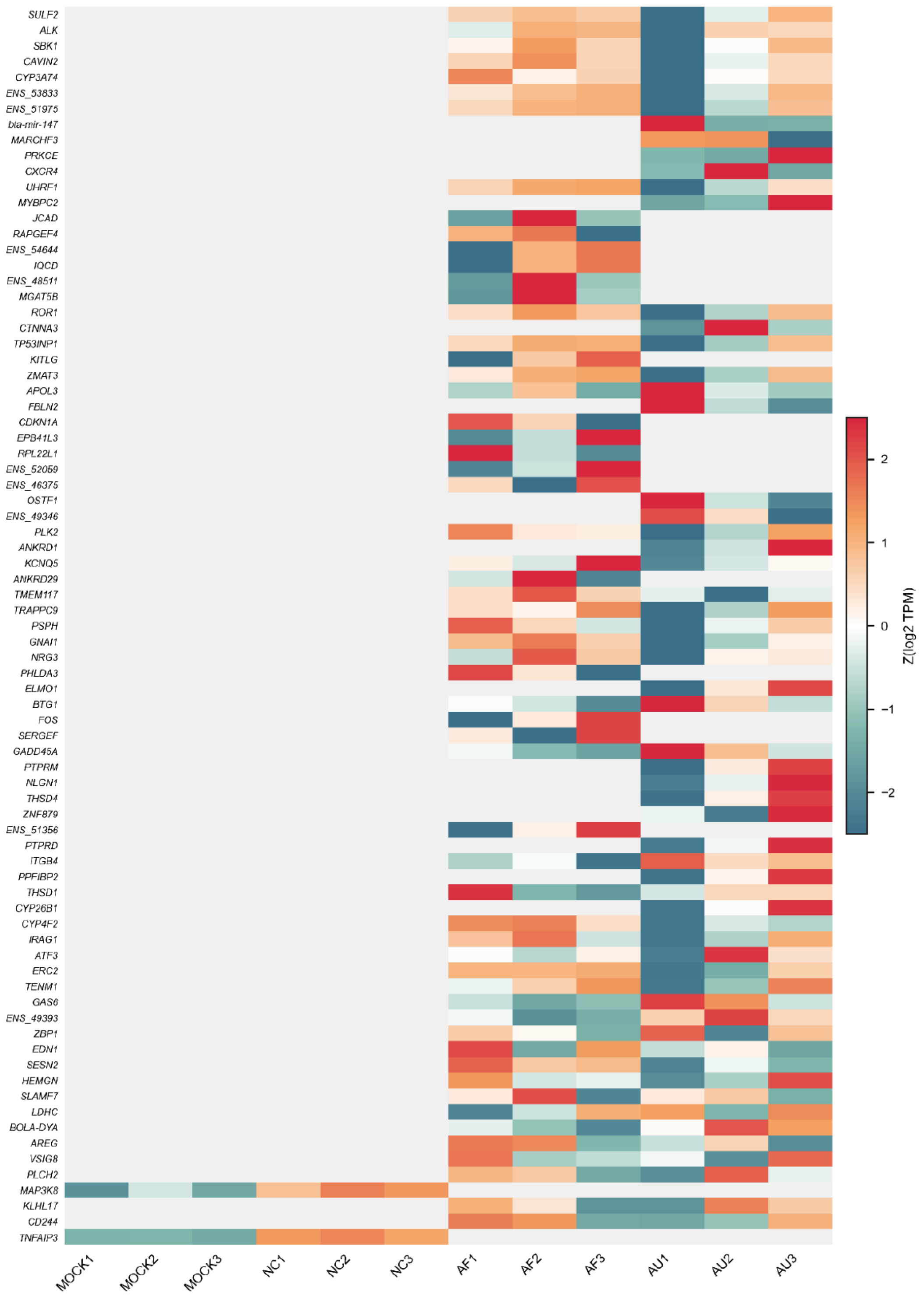

Supplementary Figure S3. Heat map of candidate-gene expression across the twelve RNA-seq libraries. Rows are candidate genes and columns are individual libraries (MOCK1–3, NC1–3, AF1–3 and AU1–3). Colour encodes the row-wise Z-score of $\log_2$-transformed TPM values; light grey indicates genes with no value available (NA) in the corresponding comparison.

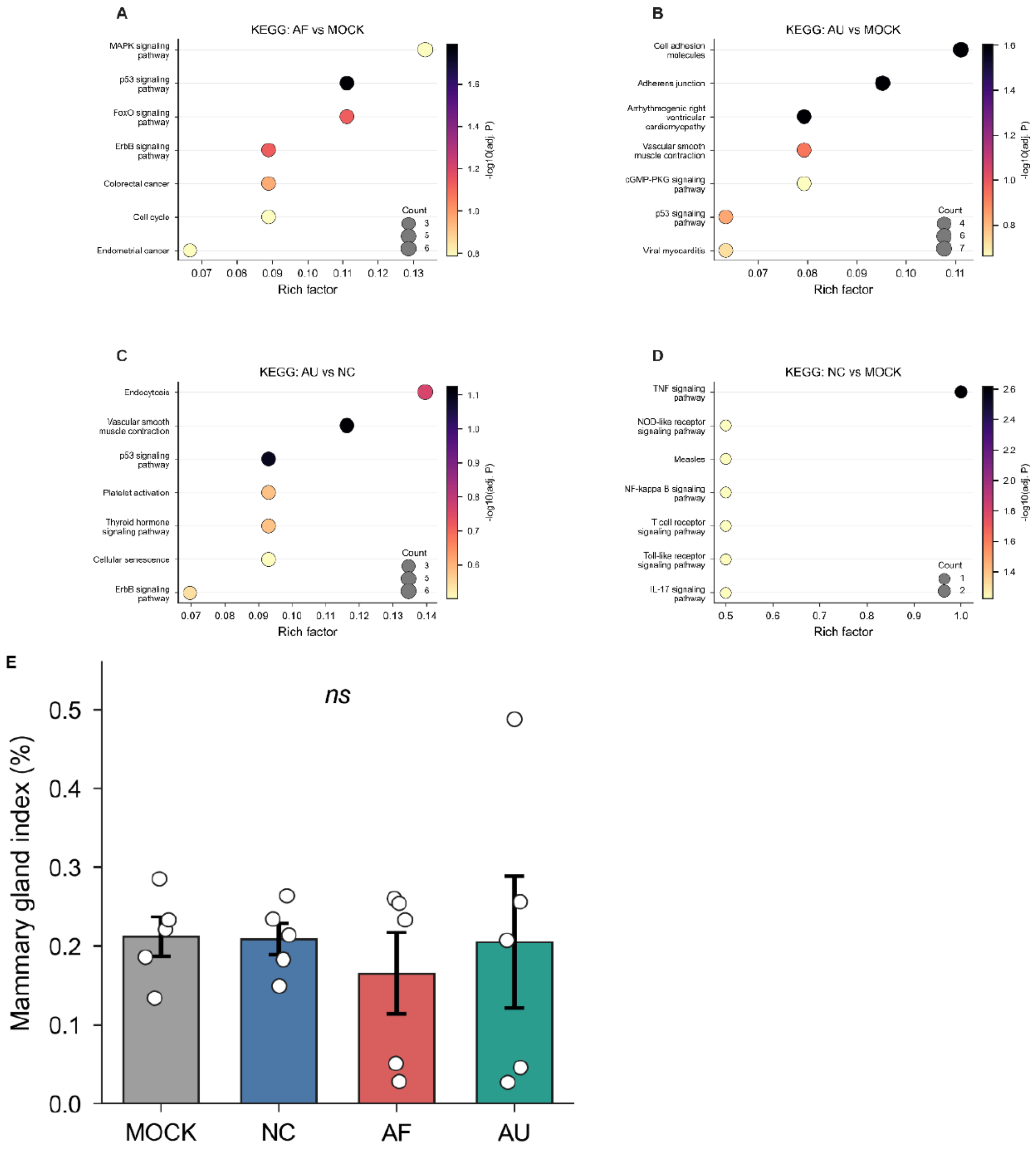

Supplementary Figure S4. Exploratory KEGG enrichment context and mammary gland index. (A–D) Exploratory KEGG pathway enrichment for AF versus MOCK, AU versus MOCK, AU versus NC, and NC versus MOCK, respectively. Bubble size represents the number of mapped genes (Count), colour represents $-\log_{10}$(adjusted p), and the x-axis shows the rich factor. (E) Mammary gland index in the MOCK, NC, AF and AU groups (n = 5 dams/group). Bars show mean ± SE, and open circles show individual dams. ns, not significant (Welch one-way ANOVA followed by Tamhane's T2; $p > 0.05$).
